# Supplementary figures and images for: Antimicrobial Resistance in Enterobacterales Recovered from Urinary Tract Infections in France
Source: Pathogens. 2022 Mar 15;11(3):356. doi: 10.3390/pathogens11030356 (PMC8949168; doi:10.3390/pathogens11030356)

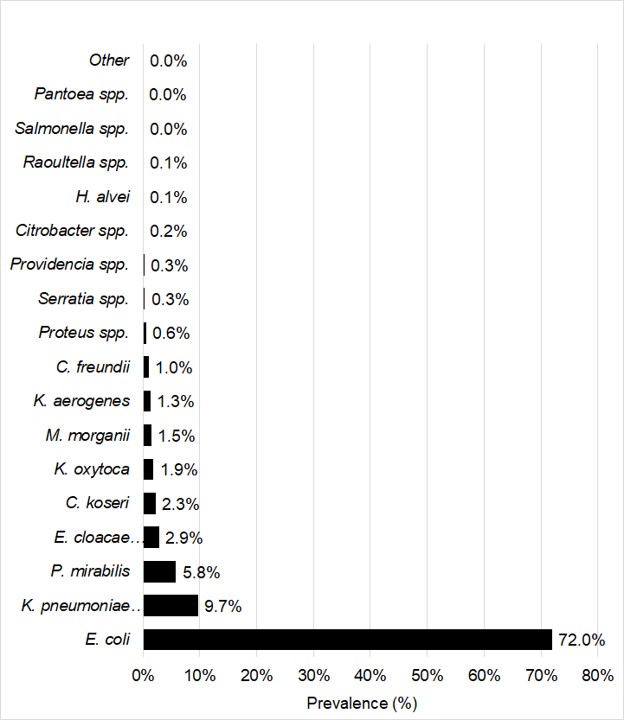

Supplement: Supplementary file 1 [file pathogens-11-00356-s001.zip › Figure_S1.jpg]

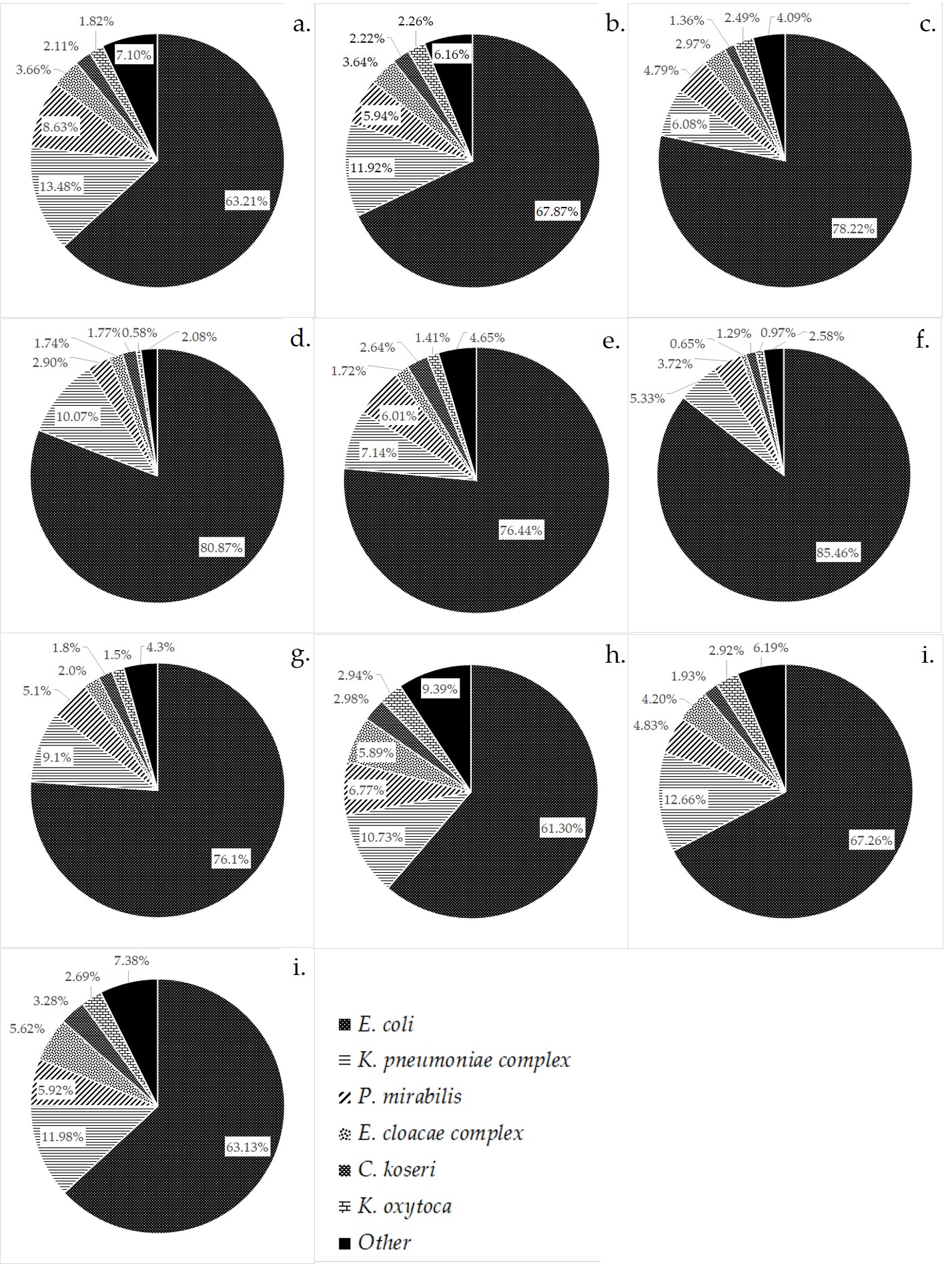

Supplement: Supplementary file 1 [file pathogens-11-00356-s001.zip › Figure_S2.jpg]

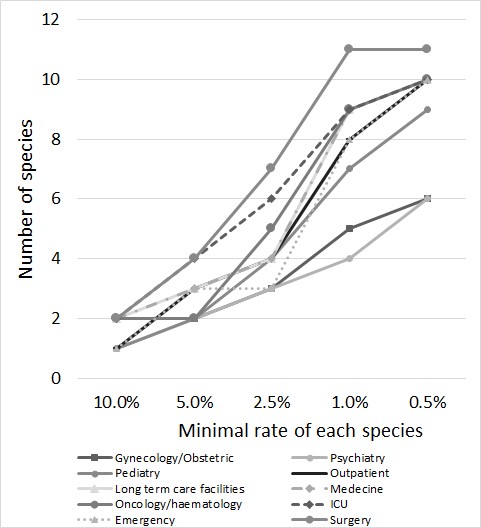

Supplement: Supplementary file 1 [file pathogens-11-00356-s001.zip › Figure_S3.jpg]

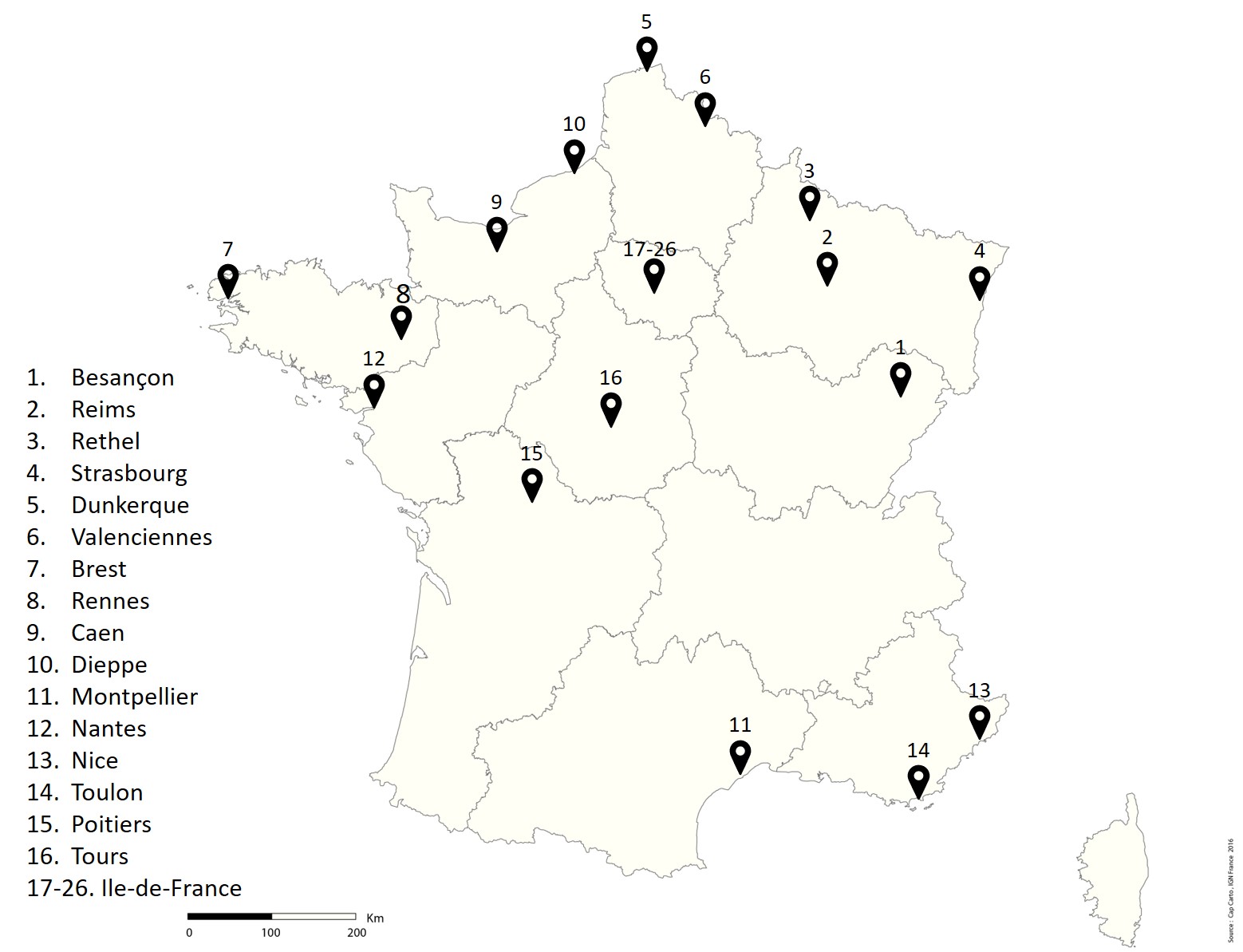

Supplement: Supplementary file 1 [file pathogens-11-00356-s001.zip › Figure_S4.jpg]
